# Significant interactions in infant operculum regions when exposed to a bilingual environment: a resting-state fNIRS study

**DOI:** 10.1117/1.NPh.12.4.045012

**Published:** 2025-12-10

**Authors:** Neda Abdollahpour, Nabi Sertac Artan

**Affiliations:** New York Institute of Technology, Department of Electrical and Computer Engineering, New York, United States

**Keywords:** functional near-infrared spectroscopy, effective connectivity, bilingualism, resting state

## Abstract

**Significance:**

Understanding the regional functionality during early development has significance across various domains such as developmental disorders.

**Aim:**

We aim to investigate the impact of early bilingual exposure on infant brain activity at the age of 4 months and to determine whether differences exist in the activity of specific brain regions between monolingual and bilingual infants in rest.

**Approach:**

To reach that aim, we utilize a combination of functional near-infrared spectroscopy and effective connectivity analysis (EC), integrated with our previously proposed graph construction method, importance of channel based on EC (ICEC), to assess neural mechanisms underlying bilingualism in infancy. Importantly, we represent a secondary analysis of a publicly available dataset.

**Results:**

Employing group-level analysis techniques, our findings reveal that bilingual experience is associated with anatomically specific rather than widespread alterations in EC. Differences were most pronounced in the superior frontal gyrus, superior temporal gyrus, and opercular regions, with the frontal and temporal cortices primarily acting as sources and the operculum functioning as both sources and sinks. Notably, bilingual infants exhibited a gradual increase in connectivity within the rolandic operculum during rest. Temporal analyses further indicated that early rest was marked by stronger inflow into right frontal–opercular hubs, whereas later rest showed a redistribution toward temporal and opercular regions with increased outflow. Together, these results provide evidence that bilingual exposure reorganizes infant brain connectivity in anatomically specific and temporally dynamic ways.

**Conclusion:**

These findings provide novel insights into the neurobiological foundations of early bilingual exposure, highlighting distinct patterns of EC in bilingual infants.

## 1 Introduction

Language experience in early life plays a crucial role in shaping both cognitive function and brain architecture. The acquisition of second or multiple languages, i.e., bilingualism or multilingualism, is increasingly recognized as a distinctive hallmark of human cognition, associated with functional and structural neuroplasticity.^1^ A fundamental feature of bilingual cognition is the simultaneous activation of more than one language, necessitating control mechanisms to manage linguistic interference.^2^ Exposure to this cognitive demand during development is believed to influence the maturation of executive functions and alter neural processing pathways.^2^ Understanding how bilingual environments impact the developing brain is therefore essential to uncovering how experience interacts with early neurodevelopment.

Infancy represents a period of exceptional neural plasticity, during which the brain rapidly adapts to environmental stimuli.^3^ Early identification of how external factors, such as language exposure, interact with this sensitive period is pivotal not only for understanding the emergence of cognition, emotion, and behavior but also for informing early diagnosis of developmental disorders, including autism spectrum disorder and language impairments.^4^^–^^6^ However, studying infant brain function presents unique methodological challenges. Although modalities such as functional magnetic resonance imaging and magnetoencephalography (MEG) offer high spatial resolution, their application in infant populations is limited due to motion sensitivity and other constraints, including sensory sensitivity to noise, the need for strict immobilization, and high operational costs and limited accessibility.^7^^,^^8^ Functional near-infrared spectroscopy (fNIRS), by contrast, is a portable, noninvasive neuroimaging modality well suited for infant research.^9^^–^^11^

fNIRS is a noninvasive optical imaging technique that measures hemodynamic responses in the cerebral cortex by detecting changes in oxygenated and deoxygenated hemoglobin (HbO/HbR) concentrations. Using near-infrared light, fNIRS provides a safe, portable, and motion-tolerant method for monitoring brain function, offering high spatial and relatively high temporal resolution.^12^^,^^13^ These characteristics have made fNIRS an increasingly valuable tool in developmental neuroscience, particularly for studying infant populations.^12^^,^^14^ Unlike other neuroimaging modalities that require stillness or sedation, fNIRS allows researchers to investigate early cognitive and social processes in awake and naturally behaving infants.^15^

To gain deeper insight into how different brain regions interact during these processes, researchers often go beyond measuring activity alone and examine how regions influence one another. This is where the concept of effective connectivity (EC) becomes central. EC refers to the directed influence that one neural region exerts over another and is central to understanding the causal architecture of brain networks.^16^ Unlike functional connectivity (FC), which quantifies undirected statistical correlations or dependencies between neural signals, EC captures the directional and potentially mechanistic relationships between brain regions.^17^ This distinction is critical as FC can suggest that regions are functionally linked without clarifying the nature or flow of information between them. EC, by contrast, enables the identification of neural pathways that actively drive responses in other regions, offering deeper insight into how the brain dynamically organizes its activity in response to different cognitive or behavioral states.^18^ By modeling EC, researchers can determine not only which areas are active but also how they interact to coordinate complex functions, allowing for a more refined interpretation of neural activity patterns across different conditions.

In this context, the present study poses the following research questions: Does exposure to an early bilingual environment impact infant brain activity in total (resting-state analysis)? More specifically, does early bilingual exposure influence the EC patterns of the infant brain, and can the proposed importance of channels using effective connectivity (ICEC) method, a novel graph-based approach, provide more detailed insights to uncover directional information flow differences between monolingual and bilingual infants?

### 1.1 Background and Related Studies

Over the past two decades, a growing body of research has investigated how bilingual environments influence infant brain development, using various neuroimaging and behavioral techniques. Among the earliest approaches, electroencephalography (EEG) has been employed to examine the neural dynamics of language processing. Studies using EEG have revealed that bilingual experience influences the organization of brain activity related to language, with evidence of enhanced phonetic discrimination and attentional engagement even in the first year of life.^19^^–^^21^ For example, Garcia-Sierra et al.^20^ and Nacar Garcia et al.^21^ demonstrated distinct patterns of neural activity in response to speech stimuli in bilingual infants, supporting the notion that early language experience modulates auditory processing at the neural level.^22^

In parallel, behavioral paradigms such as eye-tracking and attentional cueing tasks have provided additional insights into the cognitive advantages associated with bilingual exposure. These studies have reported enhanced attentional control, switching, and orienting abilities in bilingual infants compared with their monolingual peers, as seen in Refs. 23–26. Such findings suggest that bilingualism not only affects language-related processes but also promotes broader cognitive flexibility during infancy.

MEG has further supported this view, offering high temporal resolution and moderate spatial resolution of neural responses. Ferjan Ramírez et al.^27^ identified activation patterns extending into prefrontal and orbitofrontal regions, underscoring the involvement of higher-order executive areas in bilingual infants. Although these methods offer valuable insights, they are limited either by spatial coverage, as in EEG, or by practicality in infant populations, as in MEG.^28^

Recent fNIRS studies have advanced our understanding of how bilingualism shapes early brain connectivity. Blanco et al.^29^ used resting-state fNIRS (rs-fNIRS) to investigate whether monolingual and bilingual 4-month-old infants differ in their intrinsic brain network organization. Surprisingly, they found no significant group-level differences in resting-state FC patterns, suggesting that early exposure to two languages does not alter the establishment of core brain networks at this stage. Building on this, Blanco et al.^30^ performed another study to investigate whether early bilingual exposure influences resting-state functional connectivity (RSFC) in the infant brain using fNIRS. A large cohort of 4-month-old monolingual and bilingual infants (N=99) was examined during natural sleep, applying data-driven group-level analysis methods, temporal group ICA (tGICA), and connectome-based ICA (connICA), to extract functional networks and connectivity components. The analyses revealed robust and symmetric large-scale cortical networks, including auditory and language-related regions, but found no statistically significant differences in RSFC between monolingual and bilingual infants. These results suggest that the intrinsic functional organization of the infant brain may not yet reflect adaptations due to bilingual experience at this early developmental stage, though trends in language-related networks hint at potential emerging effects that may become detectable later or during active linguistic processing.

Complementing this, Blanco et al.^31^ in another study reported that bilingual infants exhibit bilateral frontal-temporal engagement during speech processing, whereas monolinguals show more left-lateralized activation. These findings imply that bilingualism may begin to shape both the spatial distribution and functional organization of language-related regions in infancy.

In parallel, Gao et al.^32^ investigated hemispheric lateralization in 4-month-old infants from different language environments using rs-fNIRS. Beyond undirected FC, they employed Granger causality (GC)-based EC analysis to examine directional communication between hemispheres. Although monolingual infants showed no strong directional connectivity, bilingual infants exhibited significant left-to-right information flow, indicating that bilingual experience may promote early development of directional cortical interactions.

The present study builds directly on these findings by utilizing the same open-access rs-fNIRS dataset of 4-month-old monolingual and bilingual infants,^30^ which provides a rare opportunity to examine the earliest stages of network formation prior to substantial language acquisition. The 4-month age point is particularly informative as it captures a developmental window where neuroplasticity is high, but environmental exposure is still relatively constrained. This makes it ideal for isolating early influences of bilingualism on brain organization.

To probe early connectivity patterns in greater detail, we apply our previously introduced graph-based method, ICEC, which quantifies both the directed adjacency edges among nodes and the strength of EC interactions for each node. By leveraging this advanced approach on infant resting-state data, we aim to uncover subtle, region-specific differences in EC between bilingual and monolingual infants. This may offer novel insights into how early auditory environments influence the developing brain. Together, these methodological and analytical advances contribute to a more nuanced understanding of infant brain organization shaped by early language exposure.

### 1.2 Research Gap and Objectives

Despite increasing interest in how bilingual environments shape infant brain development, several limitations persist in the current literature. Most prior studies have relied on FC analyses, which capture statistical relationships between regions but do not reveal the direction or causality of neural interactions. Although studies such as those by Blanco et al.^29^^,^^30^ have demonstrated robust and reproducible FC networks in 4-month-old infants, they reported no significant group-level differences between monolingual and bilingual infants. This raises the question of whether traditional FC measures are sensitive enough to detect early language-related neural differences.

To address this, Gao et al.^32^ introduced EC analysis, which offers insight into the directionality of neural interactions. Their findings revealed a left-to-right flow of information in bilingual infants, suggesting that EC may be more sensitive to experience-dependent developmental effects. However, their approach was limited in spatial resolution as it only included channels from frontal brain regions and did not account for the relative importance of specific brain regions within the network.

Thus, there remains a critical gap in understanding how bilingual exposure affects the directional and regional organization of infant brain networks, particularly at the resting state and during the preverbal period. Moreover, there is a need for advanced EC methodologies that can provide finer-grained insights into the architecture and dynamics of early brain connectivity. The present study addresses this gap by integrating a novel graph-based EC method, ICEC, which allows us to quantify both the influence and role of individual brain regions, as well as the directional flow of information between them in monolingual and bilingual 4-month-old infants.

## 2 Methods

### 2.1 Data Description

An open-access resting-state fNIRS dataset^33^ was used for this investigation. The dataset contains recordings of 99 4-month-old infants from three cohorts based on their linguistic backgrounds: 36 Basque–Spanish bilingual infants (21 females; mean age = 125 ± 4 days), 30 Spanish monolingual infants (13 females; mean age = 123 ± 3 days), and 33 Basque monolingual infants (17 females; mean age = 122 ± 4 days). Infants were positioned in a resting state, typically leaning on their parent’s laps. The spontaneous hemodynamic activity of infants’ brains during sleep, without specific tasks, was measured for more than 9 min. In line with the criteria reported in the original dataset publication, infants were classified into bilingual and monolingual groups based on parental responses to a detailed language exposure questionnaire. The bilingual group included infants who were exposed to both Spanish and Basque from birth, with more than 10% exposure to the nondominant language. Monolingual infants had either exclusive exposure to a single language or less than 10% exposure to a second language. These groupings reflect naturalistic home environments and ensure consistent classification across participants.^33^

A NIRScout fNIRS system (NIRx Medical Technologies, Orlando, Florida, United States) was used for the fNIRS measurements. The NIRScout is a continuous wave (CW) fNIRS system using light sources at two wavelengths (760 and 850 nm) with a sampling frequency of 8.93 Hz. This system consists of 24 detectors and 16 light emitters, collectively providing 52 channels for monitoring oxygenated and deoxygenated hemoglobin levels (HbO and HbR). Optode placement followed a standardized layout on a cap (Easycap GmbH, Wörthsee, Germany), conforming to the international 10 to 20 system.^34^ The channel coordinates were calculated based on the approximate optode locations, as described in Ref. 33. Each channel’s coordinates are computed as the approximate midpoint between the optode pair coordinates. The brain regions corresponding to these channels are listed in Table 1. Subsequently, channels within the occipital region of the brain (channels 47 to 52) were excluded due to the presence of low-quality signals.^30^^,^^33^ The dataset used in this study is openly available via the OSF repository shared by Blanco et al.^33^

**Table 1:** Channels and the corresponding brain regions. Each channel’s location is estimated from the source and detector placements.

Channel	Region	Channel	Region
1	Frontal sup. (L)	27	Frontal inf. tri. (R)
2	Frontal sup. (L)	28	Frontal mid. (R)
3	Frontal mid. (L)	29	Frontal mid. (R)
4	Frontal inf. tri. (L)	30	Frontal inf. oper. (R)
5	Frontal mid. (L)	31	Frontal mid. (R)
6	Frontal inf. tri. (L)	32	Frontal inf. oper. (R)
7	Insula (L)	33	Precentral (R)
8	Frontal inf. tri. (L)	34	Postcentral (R)
9	Frontal inf. oper. (L)	35	Temporal sup. (R)
10	Frontal mid. (L)	36	Postcentral (R)
11	Rolandic oper. (L)	37	Rolandic oper. (R)
12	Temporal sup. (L)	38	Postcentral (R)
13	Postcentral (L)	39	Supramarginal (R)
14	Rolandic oper. (L)	40	Temporal sup. (R)
15	Postcentral (L)	41	Supramarginal (R)
16	Postcentral (L)	42	Temporal sup. (R)
17	Temporal sup. (L)	43	Angular (R)
18	Postcentral (L)	44	Temporal mid. (R)
19	Temporal mid. (L)	45	Temporal mid. (R)
20	Supramarginal (L)	46	Temporal mid. (R)
21	Fusiform (L)	Excluded	Parietal inf. (L)
22	Temporal mid. (L)	Excluded	Occipital sup. (R)
23	Occipital mid. (L)	Excluded	Cuneus (L)
24	Frontal mid. (R)	Excluded	Cuneus (R)
25	Cingulum mid. (R)	Excluded	Occipital sup. (L)
26	Frontal mid. (R)	Excluded	Occipital mid. (R)

### 2.2 Preprocessing

**Fig. 1:**
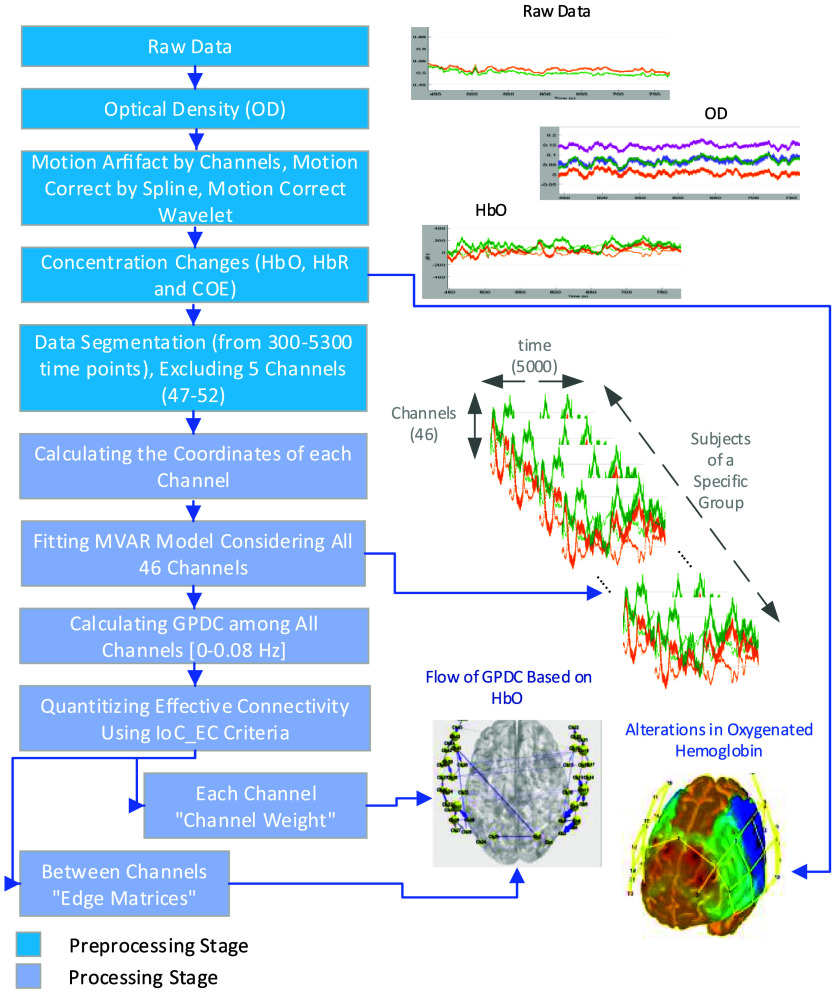
Flowchart illustrates the preprocessing (blue color) and processing (gray color) stages employed in the study. Initially, raw data are collected and converted to optical density (OD). Motion artifacts are corrected by channels using a spline and motion correction wavelets. Subsequently, changes in concentration (HbO, HbR, and COE) are computed. The data are then segmented (from 300 to 5300 time points), excluding five channels (47 to 52). The coordinates of each channel are calculated next. A multivariate autoregressive (MVAR) model is fitted considering all 46 channels. The generalized partial directed coherence (GPDC) among all channels within the frequency range of 0 to 0.08 Hz is then calculated. Finally, effective connectivity is quantified using the ICEC criteria, resulting in the determination of each channel’s “channel weight” and the “edge matrices” between channels.

The data preprocessing and processing pipeline used in this work are given in Fig. 1. In the preprocessing phase, optical density (OD) data are calculated from the light intensity data for each subject using Eq. (1), where Φ is the detected light intensity, λ is the wavelength, and i and j are the source and detector position, respectively.^35^ Following this conversion, a series of noise reduction techniques outlined by Vergotte and Torrewere are applied.^36^ Specifically, *Motion_Artifacts_by_Channel* was implemented with parameters set as tMotion=0.5  s, tMask=2  s, STDVthresh=20, and AMPthresh=0.5. Subsequently, both *Spline_MotionCorrection* and *Wavelet_Motion_Correction* methods were employed.

$$\Delta {\mathrm{OD}}_{ij(t)}^{\lambda }=\ln(\frac{\Phi_{ij}^{\lambda }(0)}{\Phi_{ij}^{\lambda }(t)}).$$ (1)

Changes in chromophore concentrations (ΔHbO and ΔHbR) were then derived from OD values using the modified Beer–Lambert law.^37^ We should note that concentration data are not filtered due to their potential to introduce spurious connections, as previous research has cautioned.^17^^,^^36^ In this investigation, we employed ΔHbO and the change in cerebral oxygen exchange (ΔCOE)^38^^,^^39^ as the primary time-varying signals for subsequent analysis during the processing stage. ΔCOE is an indicator of vascular oxygenation variations, thereby reflecting underlying neuronal activity.^40^ ΔCOE is calculated from the changes in oxygenated and deoxygenated hemoglobin concentrations, ΔHbO and ΔHbR, respectively, in Eq. (2):

ΔCOE=ΔHbR−ΔHbO. (2)

An elevation in ΔCOE signifies a decrease in capillary oxygen content, attributed to the metabolic demands of neural cells, thus indicating a state of hypoxic vascular conditions. Conversely, a decrease in ΔCOE indicates augmented oxygenation levels within blood vessels, primarily resulting from the influx of oxygen-rich erythrocytes from arterial circulation. Along with traditional markers such as ΔHbO or the changes in total hemoglobin ΔHbT, ΔCOE also offers another efficient criterion for evaluating heightened brain functionality in some studies.^39^^,^^39^^,^^41^^–^^43^

The output of the preprocessing stage consists of ∼560  s (samples 300 to 5300) of raw data comprising ΔHbO and ΔCOE measurements for each participant, consistent with prior research on this dataset.^40^ The initial and last data samples are excluded due to the susceptibility of the initial and final segments of resting-state signals to contamination from noise and movement artifacts. In addition, channels 47 to 52 located within the occipital brain region were omitted from the analysis for all subjects as these channels are deemed noisy in the original study.^33^

### 2.3 Multivariate Autoregressive Model

In this study, we employed a multivariate autoregressive (MVAR) model fitted onto a 4D tensor for each group with dimensions (1) HbO time series, (2) COE time series, (3) channels, and (4) subjects. Subsequently, generalized partial directed coherence (GPDC) was computed using (6). To compute the 4D GPDC matrices for each group, we employed EEGLAB (version 2021.1)^44^ and MATLAB (see Sec. 2.7 for suitability of EEGLAB for processing fNIRS signals).

The selection of model order, p, is critical for fitting an MVAR model. Indeed, the accuracy of model fitting hinges on the appropriate determination of model order. Too small or too large model orders can lead to inadequately detailed spectra or the generation of spurious peaks in the resulting spectrum, respectively.^45^^,^^46^ The stability test^17^ is used to ensure the reliability of connectivity estimates, model consistency, and robustness of the results. In our group-level analysis, we evaluated orders with AIC across all groups to satisfy the stability test for all samples.

In this study, we used an adaptive multivariate autoregressive (AMVAR) model, an MVAR model that can adapt to changes in the underlying process, allowing the model to handle nonstationary data where statistical properties may change over time. The Vieira-Morf algorithm^47^ with a window size of 100 and a step size of 1 is used to fit the AMVAR model. These parameters were chosen to align with our targeted frequency ranges of 0 to 0.08 Hz for this study.

### 2.4 Effective Connectivity Analysis with Generalized Partial Directed Coherence

GC provides a statistical framework to explore causal relationships between signals, which relies on linear regression modeling of stochastic processes.^45^ GC applied to an MVAR model of multisite electrophysiological recordings offers a promising avenue for identifying such connections. These methods can effectively estimate how past activity in one or more brain regions predicts current activity in others while accounting for the influence of intervening regions.^17^^,^^48^ There are several extensions of GC, including partial directed coherence (PDC),^49^ which normalizes terms in the frequency domain by the total outflow at a node. PDC lacks scale invariance; thus, arbitrary alterations in the amplitudes of individual time series can significantly influence PDC values. GPDC was introduced to ensure scale invariance within the PDC framework.^17^ Elements of the GDPC matrix can be calculated in Eq. (6)

$$X(t)=\overset{\rho }{\underset{k=1}{\sum }}A^{\left(k\right)}(t)X(t-k)+E(t),$$ (3)

$$A(f,t)=-\overset{\rho }{\underset{k=0}{\sum }}A^{\left(k\right)}(t)e^{-i2\pi fk},\;A^{\left(0\right)}=I,$$ (4)

$$X(f,t)=A(f,t{)}^{-1}E(f,t)=H(f,t)E(f,t),$$ (5)

$${\overset{¯}{\pi }}_{ij}(f)=\frac{\frac{1}{\Sigma }A_{ij}(f)}{\sqrt{\overset{M}{\underset{k=1}{\sum }}\frac{1}{\Sigma^{2}}|A_{kj}(f){|}^{2}}}$$ (6)

$$0\leq |{\bar{\pi }}_{ij}(f){|}^{2}\leq 1,\sum_{j=1}^{M}|{\bar{\pi }}_{ij}(f){|}^{2}=1,$$

where X(f), E(f), H(f), A(f), M, and ρ in Eqs. (36) correspond to the Fourier transforms of the data, residuals, transfer function, system matrix, number of variables, and the model order, respectively.^47^ Σ is the noise covariance matrix.

We use Akaike’s Information Criterion (AIC) to ascertain the optimal model order. AIC is usually preferred over other techniques such as Hannan–Quinn’s Criterion, and Bayesian–Schwartz’s Information Criterion (BIC).^17^ AIC is given by

$$\mathrm{AIC}(\rho )=\ln(|\det(V)|+\frac{2}{N}\rho K^{2}).$$ (7)

The first term depends on the estimated residual variance V(ρ) for a given ρ, whereas the second term is determined by the model order ρ, the number of channels k, and the number of data points N.^17^^,^^44^ We computed GPDC using frequency ranges from 0 to 0.08 Hz, with a step size of 0.01 Hz as these are effective frequency ranges for connectivity analysis of fNIRS signals.^36^

### 2.5 Phase-Randomized Surrogates, Edge Gating, and Statistical Analysis

As suggested in prior work, synthetic data can validate connectivity methods in fNIRS.^50^ In this study, EC was estimated with EEGLAB’s implementation of an MVAR (AMVAR) model and GPDC. ICEC does not generate interactions; it quantifies and ranks connections derived from these validated EC estimates. EC was estimated per subject. To suppress spurious connections, we applied EEGLAB’s phase–randomization surrogates (1000 surrogates per subject; two–tailed, α=0.05). For each directed edge, the observed GPDC was compared with the subject-specific surrogate distribution; multiplicity was then controlled within subject using Benjamini–Hochberg FDR at q=0.05 across the full edge set, and only FDR-surviving edges were retained to compute node-level metrics (ICEC).

For group inference, we analyzed both group (bilingual versus monolingual) and status (bilinguals, Spanish monolinguals, Basque monolinguals) across 0.02 to 0.06 Hz using a multivariate GLM. For each channel, four dependent variables were entered: HbO-to (sink/inflow), HbO-from (source/outflow), COE-to (sink/inflow), and COE-from (source/outflow). Overall multivariate effects were assessed with MANOVA (Pillai’s Trace, Wilks’ Lambda, Hotelling’s Trace, Roy’s Largest Root), followed—when appropriate—by univariate ANOVAs. Estimated marginal means (EMMs) were used for interpretation; pairwise comparisons were Bonferroni-adjusted within channel, and BH-FDR at q=0.05 was applied across channels for each dependent variable to control the expected false discovery rate at the channel level.

### 2.6 Importance of Channels Using Effective Connectivity

The proposed ICEC framework builds upon our previously validated and benchmarked method.^51^ The reliability and effectiveness of the proposed ICEC method were evaluated using three benchmark EEG datasets from BCI Competitions III and IV, ensuring consistency across different subjects and recording setups. The method was compared with several state-of-the-art CSP-based channel selection techniques (e.g., SCSP, SFFS, IBGSA, CSP-Rank), achieving the highest mean accuracies (82%, 86.01%, and 87.56%) with significantly fewer channels and statistically significant improvements (p<0.05). Model reliability was confirmed through participant-specific MVAR stability and AIC-based model order selection, whereas consistent channel rankings and visualized connectivity maps demonstrated robustness and interpretability across frequency bands. In this work, we extend the framework and evaluate its performance under new experimental conditions.

The evaluation of ICEC for each channel encompasses five primary steps. Initially, the 4D EC matrix, B, undergoes dimensionality reduction in the first two steps, utilizing specific windows. These windows delineate the boundaries of the third (frequency) and fourth (time) dimensions, respectively. For this investigation, we selected windows encompassing all time and frequency samples for the fourth and third dimensions, correspondingly. The outcome of these initial steps yields the $N_{\mathrm{ch}}\times N_{\mathrm{ch}}$ edge matrix C, which quantifies directed interactions among channels, where $N_{\mathrm{ch}}$ represents the total number of electrodes for each task. The (i,j)’th element of C, $C_{ij}$ serves as a metric depicting the average interaction intensity between nodes i and j. $C_{ij}$ is derived from the EC matrix, B, and can be computed by

$$C_{ij}=\frac{\overset{f_{\max}}{\underset{f=f_{\min}}{\sum }}\overset{w_{\max}}{\underset{w=w_{\min}}{\sum }}{\mathrm{con}}_{ijfw}}{\left(w_{\max}-w_{\min})(f_{\max}-f_{\min}\right)}.$$ (8)

Upon sorting the quantities of $C_{ij}$ within the matrix and aggregating the highest values for each electrode, ICEC is obtained for each channel, serving as a metric to assess the significance of each electrode. The μ limit is selected as eight through iterative experimentation in this study.^51^

It is important to emphasize that ICEC is not a new estimator of EC but rather a graph-construction criterion designed to enhance the interpretability of established EC measures. As demonstrated in related applications such as fatigue detection,^52^ ICEC can serve as a powerful pattern-recognition tool across diverse domains. This criterion is applied to EC matrices derived from validated estimators such as GPDC, PDC, or DTF to extract more meaningful information from network structures. In practice, ICEC quantifies the relative importance of each channel (node) based on statistically validated connections, thereby ranking sources and sinks rather than creating new links. In this study, EC matrices were first obtained using EEGLAB implementations and subsequently subjected to surrogate data testing (phase randomization), ensuring that only genuine interactions were retained before ICEC quantification. Thus, ICEC does not introduce additional dependencies or inflate connectivity patterns but instead provides a principled means of weighting and interpreting networks already supported by statistical testing. In this way, ICEC should be understood as a graph-based analytical framework that increases the sensitivity and interpretability of EC analyses while preserving their specificity.

### 2.7 Channel Weights and Edge Matrices

In this study, we defined edge matrices that contained EC interactions between all pairs of channels using $C_{ij}$. We also put the channel weights equal to each channel’s ICEC measure. After evaluating the edge matrix and channel weights using 4D GPDC matrices for each group, the results were visualized using the BrainNet Toolbox^53^ (Version 1.7, Release 2019) in MATLAB (version 2018b). For calculating the GPDC measures, we applied EEGLAB. Although EEGLAB is primarily designed for processing EEG signals, it is suitable for analyzing other time-varying biosignals such as magnetoencephalography (MEG)^54^ and electrocardiography (ECG)^55^ in general. To the best of our knowledge, this is the first time it has been used for fNIRS. However, we encountered no issues regarding its use for this particular purpose.

## 3 Results

In this section, we (i) summarize group-specific activation patterns using EC-derived ICEC, visualized with channel-wise topoplots; (ii) characterize inter-regional connectivity across three nonoverlapping time intervals to capture the temporal evolution of interactions; and (iii) compare the spectral profiles of HbO and COE between groups using HRF-based analyses. Finally, full statistical procedures are provided in a dedicated subsection.

**Fig. 2:**
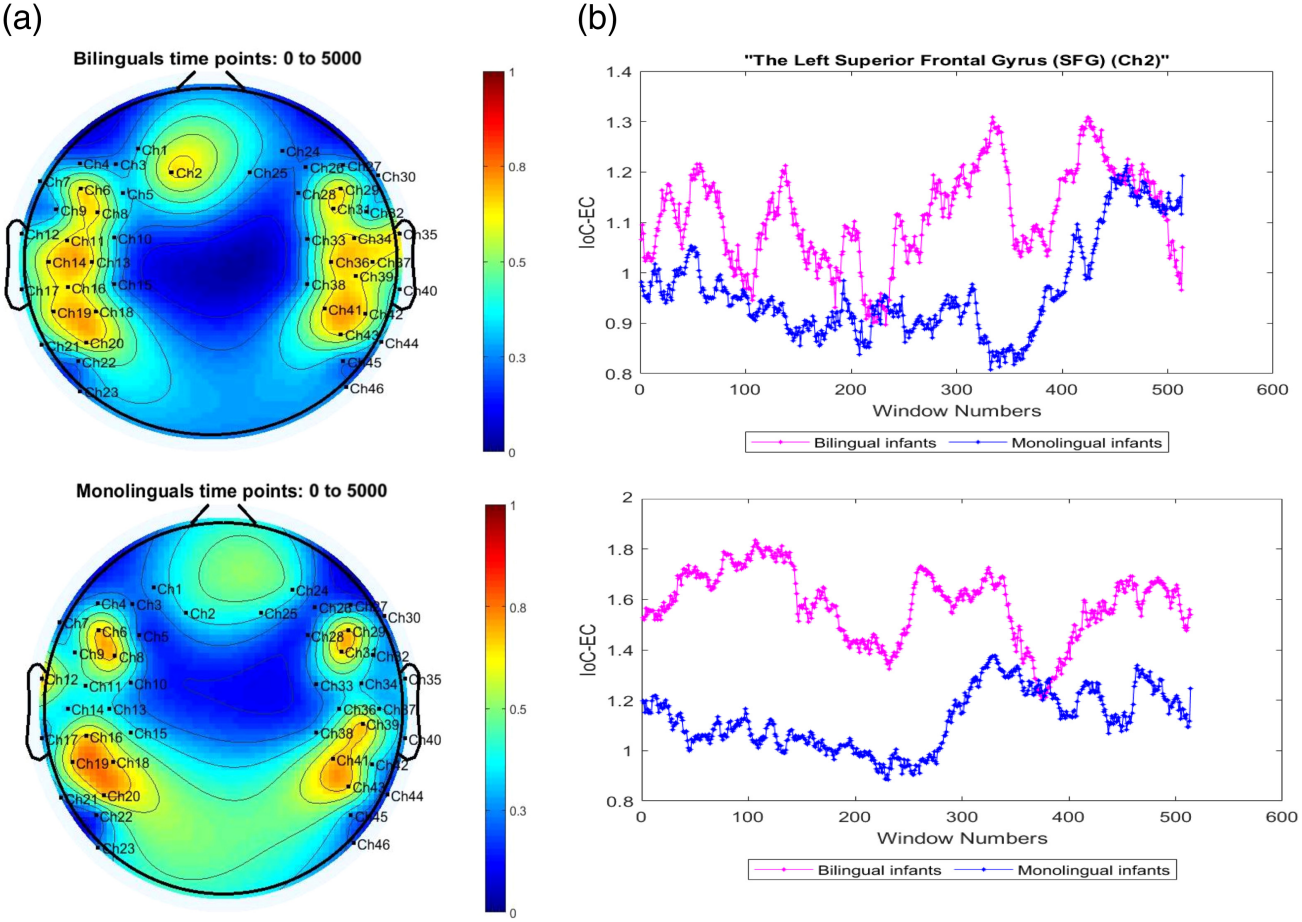
(a) Topographic plots unveil discernible disparities in neural activation patterns between cohorts of bilingual and monolingual infants. (b) The comparison of the measure of ICEC criteria based on GPDC between the two groups for channel 2 (top), and channel 11 (bottom), situated, respectively, in the SFG and the left operculum region. The x-axis indicates the window number used when calculating GPDC. Each window contains 100 time points, corresponding to a total of 514 windows.

**Fig. 3:**
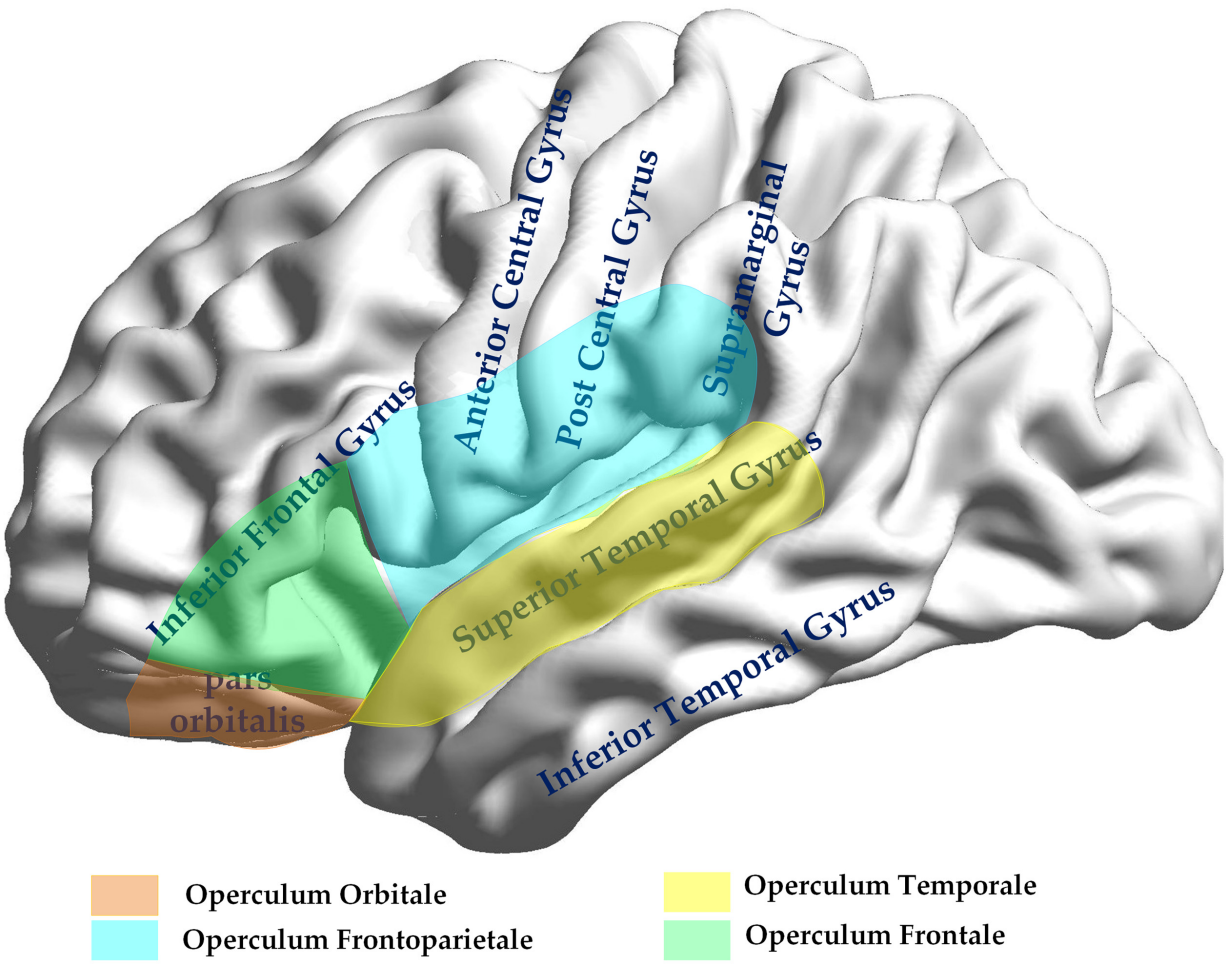
Operculum regions of the brain.

The topoplots of regional brain activity in Fig. 2(a) compare the activated brain regions in bilinguals and monolinguals. These plots show the amount of information outflow for each channel quantified using the ICEC measures. These topoplots are descriptive and illustrate apparent group differences in EC. Channels 11, 14, and 37 in opercular regions (Fig. 3) show higher activity in bilinguals, and channels 2, 33, and 34 (left/right SFG, precentral, and postcentral gyrus) also trend higher in bilinguals. For details about statistical tests and multiple-comparison procedures, see Sec. 3.1.

Figure 2(b) shows how the ICEC measures change with time through the testing period for channels 11 and 2. Accordingly, ICEC measures for bilinguals were higher for most of the time, which also demonstrates that these channels were more active based on GPDC measures for infants exposed to two languages.

**Fig. 4:**
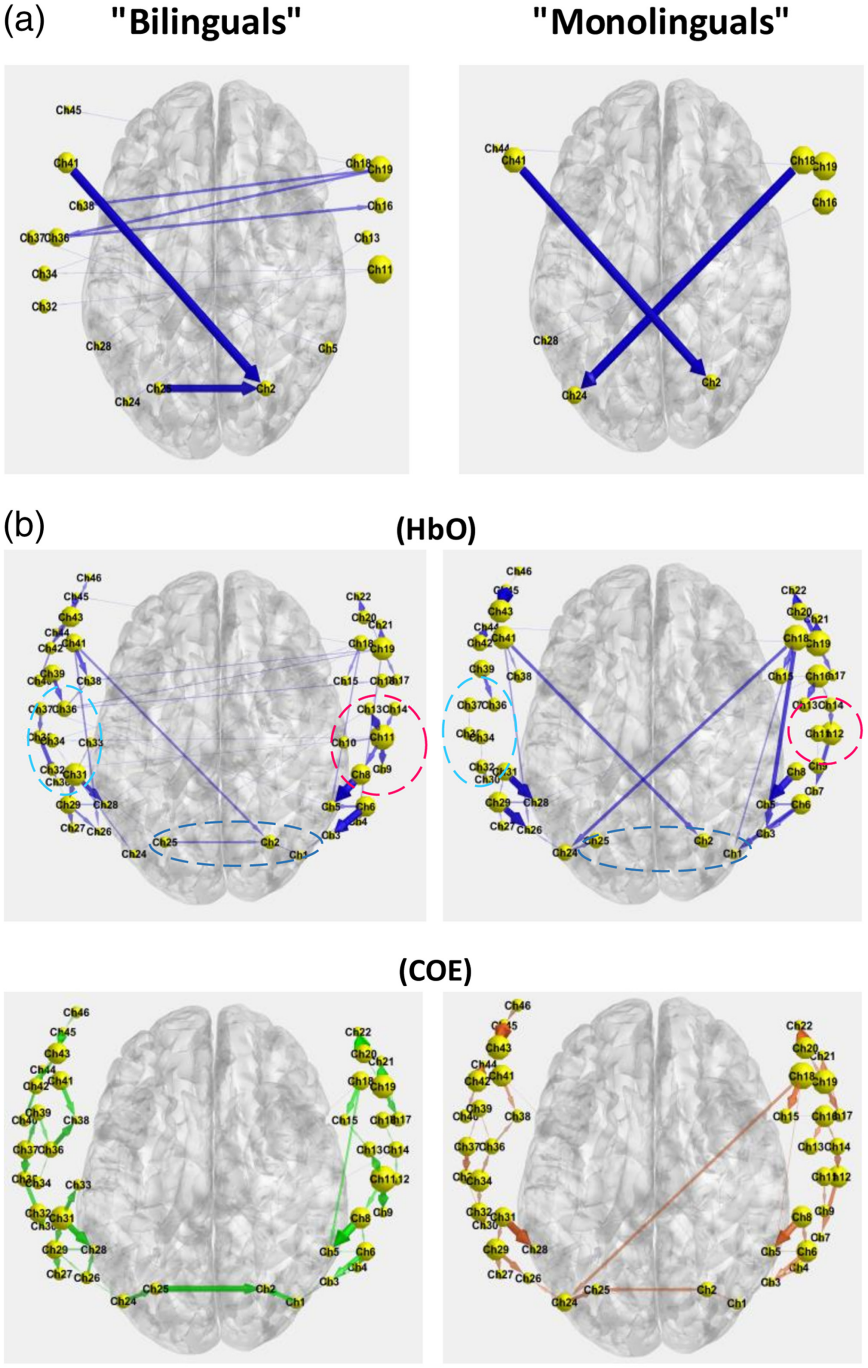
(a) Inter-hemispheric visualization of the EC for bilingual and monolingual infants. (b) The HbO-based (top) and COE-based (bottom) EC flow for both groups.

Figure 4(a) compares the inter-hemispheric EC flow between the two groups, which differ in two aspects. First, bilinguals show some interactions in the left and right Operculum, SFG, and postcentral brain regions. Second, the bilingual brains show a clear information flow from right to left in the SFG region. Conversely, both of these behaviors are absent in the monolingual group. Fig. 4(b) further illustrates EC flow for both groups for HbO (top) and COE (bottom). EC flow based on HbO from postcentral to the frontal regions is obvious for both groups. However, bilingual infants have more outflow from channels 11 and 35, and more inflow to channels 13 and 32, compared with the monolingual infants.

**Fig. 5:**
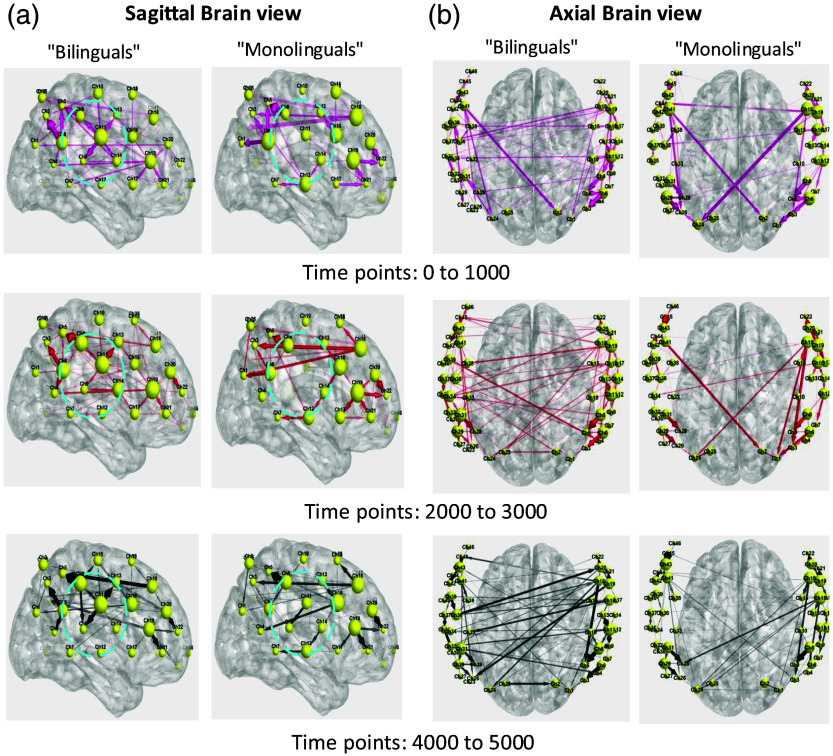
Longitudinal assessment of group results across three time intervals (0 to 1000, 2000 to 3000, and 4000 to 5000). (a) Sagittal brain view and (b) axial brain view.

To determine the differences across the time course of the study between monolingual and bilingual infants, we further analyzed the data in three distinct time intervals: 0 to 1000, 2000 to 3000, and 4000 to 5000 (Fig. 5).

Across all the time periods, channels located in the left and right operculum regions (channels 11, 14, 30, 32, and 37) exhibited consistent activity in the bilingual group, whereas activation commenced only in the final time interval for monolingual infants [dashed circles in Fig. 5(a)]. Figure 5(b) illustrates the axial view of the brain across these time intervals.

In addition, activity in the left and right Rolandic operculum regions persisted over time for the bilingual group compared with monolingual infants. Notably, ICEC measures (represented by the size of channels) keep rising over the period for both groups. Overall, bilinguals exhibited higher total interactions among channels compared with the other group.

Statistical analysis shows that during the early rest period (period 1), the right inferior frontal operculum (channels 30 and 32) and the right rolandic operculum (channel 37) exhibit higher inflow (the “to” connectivity, i.e., sink behavior). During the late rest period (period 3), the left rolandic operculum (Channel 11) shows higher inflow, whereas the right inferior frontal operculum (channel 32) shows higher outflow (the “from” connectivity, i.e., source behavior). In summary, inflow is strongest at right frontal–opercular sites early and shifts to the left rolandic region later, with channel 32 transitioning from sink to source across periods, see Sec. 3.1 for more details.

**Fig. 6:**
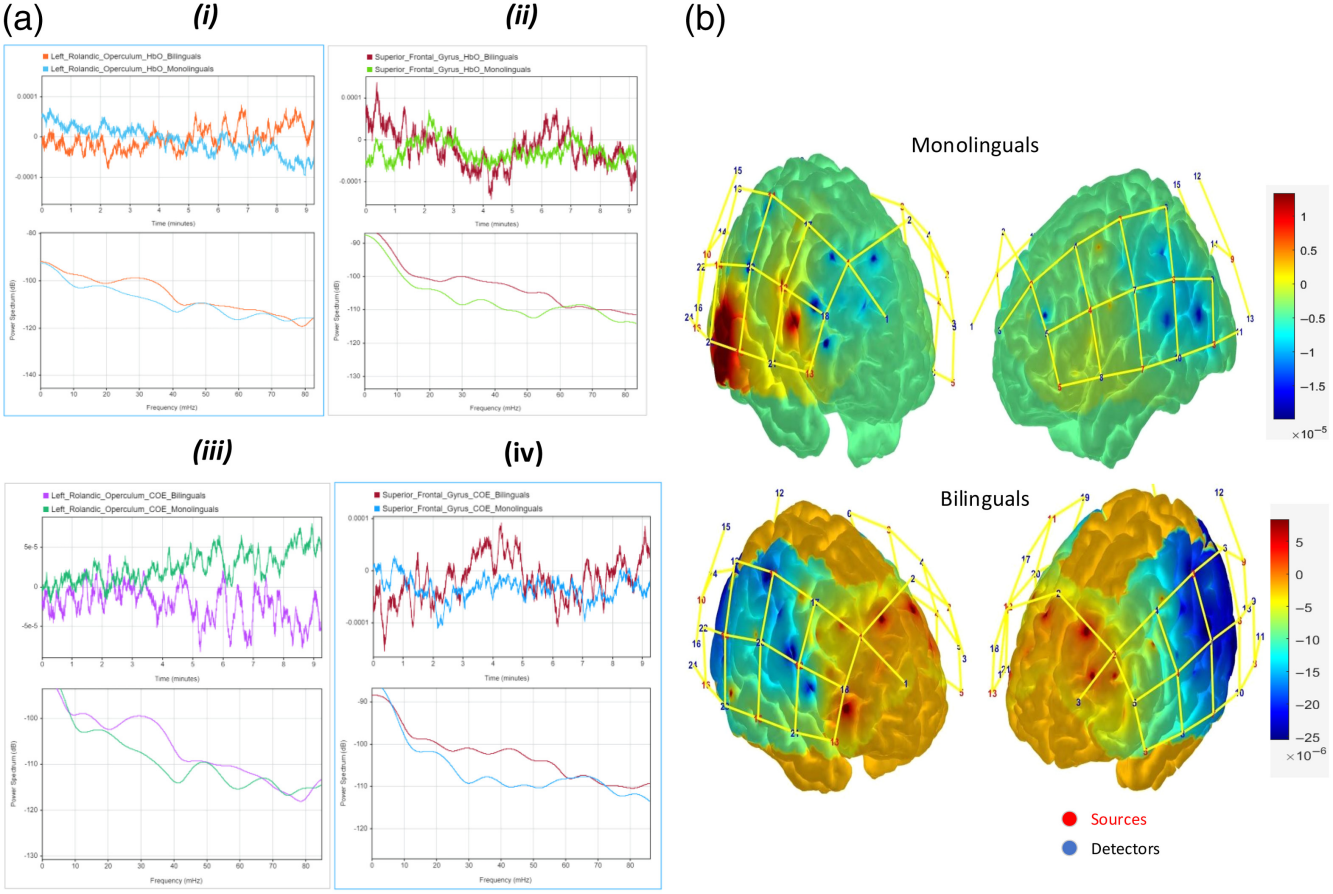
(a)(i-ii) HbO time series for two specific brain regions, namely, the Rolandic operculum and the SFG, are depicted. Correspondingly, power spectrum figures reveal notable between-group differences across various frequency ranges, notably within the range of 0.02 to 0.06 Hz. (a)(iii-iv) Analogous illustrations, albeit based on COE. (b) Changes in HbO concentration over 5000 time points during the resting-state task for both bilingual infants and monolinguals. Examination of the figures reveals heightened HbO changes in channels 42 and 44 located between source-detector pairs (S14 and D23) and (S16 and D23), respectively. In the bilingual group, HbO concentration changes in the frontal lobe are notably pronounced compared with the monolingual group. Specifically, channels such as 2, 25, 27, and 3 situated between pairs of sources and detectors (S2 and D2), (S1 and D2), (S13 and D18), and (S2 and D5), respectively, exhibit this trend.

Due to the differences observed at the Rolandic Operculum and the SFG, spectral analysis is also carried out focusing on these two regions, as shown in Fig. 6(a), which shows the time series and power spectrum for these regions based on HbO [Fig. 6(a).i-ii] and COE [Fig. 6(a).iii-iv]. This analysis highlights discernible inter-group disparities observed across a range of frequency bands.

In Fig. 6(a), differences between frequency ranges from 0.02 to 0.06 Hz in the Operculum and the SFG regions are obvious among groups.

Statistical analysis shows that in this frequency range (0.02 to 0.06 Hz), channels 2 and 12, located in the superior frontal and superior temporal cortices, emerged as principal source regions driving differential connectivity. By contrast, the inferior frontal and rolandic opercular sites (channels 30, 32, and 37) displayed a dual role, acting both as sources and sinks of information flow. Together, these findings suggest a reorganized frontal–temporal network architecture in which bilingual experience reshapes EC within language-relevant hubs. (For more details, see Sec. 3.1)

In Fig. 6(b), we used the hemodynamic response function (HRF) to depict the results. To satisfy the requirement for calculating these criteria, we intentionally add an artificial stimulus for all subjects. Examination of Fig. 6(b), generated using AtlasViewerGUI,^56^ reveals heightened changes in HbO levels between two groups. Notably pronounced changes in HbO concentration within the frontal lobe are observed in the bilingual group compared with the monolingual group, specifically, channels 2, 25, 27, and 3.

### 3.1 Accounting for HbO–COE Dependency: Group- and Status-Level Effects on Connectivity (FDR-Corrected)

To enable a more detailed examination of our results, we extended the analysis to consider all three status groups (bilinguals, Spanish monolinguals, Basque monolinguals) rather than collapsing the monolinguals. This is worth mentioning that AMVAR/GPDC is estimated per subject, and then, statistics are computed across subjects. Because HbO and COE are dependent measures, we applied a multivariate framework that explicitly models their covariance structure. To further safeguard against false positives, the entire pipeline included correction procedures for multiple comparisons.

#### 3.1.1 Frequency–wise statistical summary

We analyzed group (bilingual versus monolingual) and status (status 1: bilinguals; status 2: Spanish monolinguals; status 3: Basque monolinguals) effects across the frequency range 0.02 to 0.06 Hz using a multivariate general linear model (GLM) in SPSS. For each channel, four dependent variables were included: HbO-to (inflow), HbO-from (outflow), COE-to (inflow), and COE-from (outflow) over all subjects. Omnibus effects were first tested with MANOVA (Pillai’s Trace, Wilks’ Lambda, Hotelling’s Trace, Roy’s Largest Root), followed by univariate ANOVAs when appropriate. EMMs were used for interpretation, and pairwise comparisons were Bonferroni-corrected. To further minimize false positives, we also applied FDR correction across channels.

Pairwise comparisons revealed significant differences in HbO outflow in the left superior frontal gyrus, channel 2. Specifically, status 1 differed from status 2 (p=.009) and also differed from status 3 (p=.002). This suggests that the superior frontal gyrus acted as a differential source node across groups. In the left superior temporal gyrus, channel 12, a significant group effect was observed for COE-from connectivity (F(1,96)=5.390, p=.022, partial $\eta^{2}=.053$), with bilinguals (M=.736) exhibiting stronger outflow than monolinguals (M=.663). Pairwise comparisons further indicated that status 1 differed from status 3 on COE-from (p=.007). This positions the superior temporal cortex as a source node differentiating bilinguals from Basque monolinguals.

At channel 30 (right inferior frontal operculum), univariate analysis revealed a significant status effect for HbO-to connectivity (F(2,96)=4.605, p=.012, partial $\eta^{2}=.088$). Pairwise comparisons indicated that status 1 differed from status 2 (p=.036) and from status 3 (p=.004). Although other contrasts did not survive correction, this trend highlights the inferior frontal operculum as a potential sink node where connectivity inflow varies across language groups. In the right inferior frontal operculum (channel 32), a significant status effect was also observed for HbO-to connectivity (F(2,96)=6.139, p=.003, partial $\eta^{2}=.113$). Pairwise comparisons showed that bilinguals (M=.673) exhibited lower inflow than monolinguals (M=.724; p=.006). Additional differences emerged, with status 1 differing from status 3 (p<.001) and also status 2 (p=.045). These findings suggest that the right inferior frontal operculum functions as a sink node, with monolinguals integrating stronger inputs than bilinguals. At the right rolandic operculum, channel 37, significant group and status effects emerged at the multivariate level (p<.05). At the univariate level, HbO-from connectivity showed a robust group effect, with bilinguals demonstrating greater outflow than monolinguals (p=.002). Status effects were also evident for HbO-from (F(2,96)=5.265, p=.007, partial $\eta^{2}=.099$) and COE-to (F(2,96)=3.329, p=.040, partial $\eta^{2}=.065$). Pairwise contrasts confirmed that for HbO-form, status 1 differed from both status 2 (p=.023) and status 3 (p=.003). For COE-to, status 1 differed from status 3 (p=.012), and status 2 differed from status 3 (p=.037). This pattern identifies the rolandic operculum as both a source and sink, with bilinguals showing stronger output and monolinguals exhibiting greater integration of inputs.

Taken together, these effects were anatomically specific rather than widespread. The superior frontal and superior temporal cortices (channels 2 and 12) primarily acted as sources of differential connectivity, whereas the inferior frontal and operculum regions (channels 30, 32, and 37) functioned as both sources and sinks. These results point to a network-level reorganization in which bilingual experience modulates EC across frontal–temporal hubs relevant for language processing.

#### 3.1.2 Two-period statistical summary

(early versus late rest) Group/status differences were concentrated in frontal–opercular regions at the beginning of the resting state and shifted toward temporal/operculum sources at the end.

Early (period 1): the right inferior frontal operculum showed stronger inflow on HbO-to at Ch30 (F(2,96)=4.403, p=.015, partial $\eta^{2}=.084$; status 1 > status 2, p=.019; bilinguals > monolinguals, p=.004) and at Ch32 (F(2,96)=3.713, p=.028, partial $\eta^{2}=.072$; bilinguals > monolinguals, p=.027). The right rolandic operculum (Ch37) also showed a status effect on HbO-to (F(2,96)=3.119, p=.049; status 1 > status 2, p=.014) and a group effect (bilinguals > monolinguals, p=.028). Late (period 3): frontal inflow effects abated (Ch30 non-significant; Ch32 HbO-to non-significant), whereas a temporal/opercular pattern emerged—Ch11 showed a status effect on COE-to (F(2,96)=3.364, p=.039; status 1 > status 3, p=.015), and Ch32 showed a status effect on HbO-from (F(2,96)=4.531, p=.013; status 1 > status 2, p=.013). Other contrasts were nonsignificant.

Overall, early rest was characterized by greater inflow (sink-like behavior) into right frontal–opercular hubs in bilinguals, whereas late rest showed a shift toward temporal/operculum regions with increased outflow/COE effects and diminished frontal differences. Effect sizes were in the small–to–moderate range (partial $\eta^{2}\approx 0.06$ to 0.11). Pairwise findings without a significant omnibus (e.g., Ch11 HbO-to) should be interpreted as descriptive/exploratory.

## 4 Discussion

In this investigation, we combined EC analysis with our recently introduced ICEC framework to examine neural interaction patterns in bilingual and monolingual infants. Importantly, the EC estimates were validated through surrogate testing, ensuring that ICEC quantifies genuine interactions rather than spurious connections. This methodological advance strengthens confidence in the reliability of the observed effects and highlights the utility of ICEC for probing early developmental connectivity.

A central finding of our study was the identification of differences in opercular and superior frontal regions between bilingual and monolingual infants. To the best of our knowledge, no previous fNIRS study has reported such opercular differences in this population. In bilingual infants, the rolandic operculum in particular showed a gradual increase in connectivity during rest, suggesting that bilingual experience influences integration in regions beyond the canonical frontal hubs. This observation aligns with structural neuroimaging evidence in adults showing bilingualism-related changes in opercular pathways,^1^ thereby bridging early functional differences in infants with known long-term adaptations.

These findings invite comparison with the work of Gao et al., who analyzed the same dataset using GC and graph-theoretic approaches. Although this study converges on the conclusion that frontal information flow is present in bilingual but not in monolingual infants, important differences emerge. Gao’s analysis focused primarily on hemispheric lateralization within frontal regions, whereas our approach extended the evaluation to all available channels, thereby capturing connectivity in a more global framework.

Perhaps most notably, Gao et al. reported a left-to-right frontal flow in bilinguals, whereas our results indicate more specific inflow and outflow patterns in specific brain areas such as operculum regions. This discrepancy may reflect the influence of interactions between frontal and nonfrontal channels, which were explicitly modeled in ICEC. By accounting for both inflow and outflow at each node, as well as dependencies between HbO and COE signals,^51^ ICEC provides a more comprehensive view of how bilingual exposure shapes early neural communication.

Taken together, our results show that bilingual experience was associated with anatomically specific, rather than widespread, differences in connectivity. Frontal and temporal hubs, particularly the superior frontal gyrus, superior temporal gyrus, and inferior/rolandic opercula, emerged as the most sensitive nodes to group and status effects. The superior frontal and superior temporal cortices primarily acted as sources of differential connectivity, whereas the inferior frontal and opercular regions functioned as both sources and sinks. This division of roles suggests that bilingualism may reorganize the balance of information flow across frontal–temporal circuits, strengthening outflow from language-related sources while modulating the integrative functions of opercular sinks. Such a pattern aligns with prior neuroimaging evidence linking bilingualism to adaptive changes in executive and linguistic control networks,^57^^–^^59^ and it highlights that connectivity reorganization is regionally concentrated rather than diffuse.

Temporal dynamics further revealed that the organization of connectivity was not static across rest but shifted from early to late periods. Early rest was characterized by stronger inflow into right frontal–opercular regions in bilinguals, consistent with enhanced integration and sink-like behavior of frontal hubs. By contrast, late rest showed a redistribution of effects toward temporal and opercular regions, with increased outflow and COE-driven influences and diminished frontal asymmetries. These findings suggest that bilingual experience impacts both the spatial and temporal dimensions of EC, with frontal hubs dominating early network integration and temporal/opercular sources emerging later. Effect sizes were modest but consistent, supporting the view that bilingualism induces nuanced, time-dependent shifts in resting-state network dynamics rather than large-scale structural differences.

An important limitation of this work is that EC derived from fNIRS signals should be interpreted as “virtual” rather than direct evidence of neuronal communication. GPDC applied to hemodynamic time series provides a model-based approximation of directional influences, but it cannot fully capture the underlying physiological mechanisms. This caveat is particularly relevant in the context of 4-month-old infants during sleep, where higher-order processes such as speech perception or executive control are unlikely to be directly engaged. Nevertheless, developmental studies have shown that differences in neural organization between monolingual and bilingual infants can emerge very early, even before full perceptual narrowing occurs.^27^^,^^60^ Thus, it is plausible that early bilingual exposure shapes large-scale patterns of hemodynamic connectivity, detectable even during sleep, through mechanisms related to attentional control or early perceptual tuning. Our findings should therefore be viewed as preliminary but meaningful evidence of such early differences, providing a foundation for future studies to clarify the specific neurophysiological processes involved.

### 4.1 Implications and Future Directions

The present findings extend current bilingualism research by demonstrating that EC differences can be detected in infancy during resting state, even in the absence of explicit task demands. This underscores the sensitivity of directional connectivity measures such as GPDC for probing early neurodevelopment. Methodologically, the combination of HbO and COE in a multivariate framework, along with rigorous correction for multiple comparisons, provides a robust template for future infant fNIRS studies. Theoretically, the observed frontal–temporal reorganization suggests that bilingual exposure may shape not only localized hemodynamic responses but also the directional architecture of large-scale networks from an early age. Future work should aim to replicate these findings with larger samples, include longitudinal designs to track developmental trajectories, and examine how these early connectivity patterns relate to later cognitive and linguistic outcomes. Expanding the analysis to other frequency bands and incorporating multimodal measures (e.g., EEG–fNIRS) may also help clarify the physiological underpinnings of bilingual brain plasticity.

Together, these results provide some of the earliest evidence that bilingual experience reorganizes infant brain connectivity in anatomically specific and temporally dynamic ways, offering novel insights into how language exposure shapes neurodevelopment from the very beginning of life.

## 5 Conclusion

This study provides some of the earliest evidence that early bilingual exposure reorganizes infant brain connectivity in anatomically specific and temporally dynamic ways. Using fNIRS combined with our ICEC framework, we identified distinct connectivity patterns in frontal and opercular hubs, with bilingual infants showing increased integration in the rolandic operculum absent in monolinguals. These effects were not widespread but concentrated in key language-related regions, suggesting that bilingualism shapes the balance of sources and sinks within frontal–temporal networks even during rest at 4 months of age. Importantly, by validating ICEC estimates with surrogate testing and explicitly modeling dependencies between HbO and COE, we strengthened the robustness of EC analysis in infancy.

Together, these findings advance our understanding of how early bilingual experience influences the organization of large-scale brain networks, offering important insights into the neurobiological basis of language development. Although the results should be interpreted cautiously given the reliance on hemodynamic proxies and resting-state infant data, they nevertheless highlight bilingualism as a factor shaping early neural communication. Future work should replicate these effects in larger longitudinal cohorts, extend analyses across additional frequency bands, and integrate multimodal approaches such as EEG–fNIRS to further clarify the mechanisms of bilingual brain plasticity.

## Data Availability

The dataset used in this study is openly available via the OSF repository shared by Blanco et al.^33^ at: https://doi.org/10.17605/OSF.IO/7FZKM.
